# GPRuler: Metabolic gene-protein-reaction rules automatic reconstruction

**DOI:** 10.1371/journal.pcbi.1009550

**Published:** 2021-11-08

**Authors:** Marzia Di Filippo, Chiara Damiani, Dario Pescini

**Affiliations:** 1 Department of Statistics and Quantitative Methods, University of Milan-Bicocca, Milan, Italy; 2 Department of Biotechnology and Biosciences, University of Milan-Bicocca, Milan, Italy; 3 SYSBIO Centre of Systems Biology, Milan, Italy; The Pennsylvania State University, UNITED STATES

## Abstract

Metabolic network models are increasingly being used in health care and industry. As a consequence, many tools have been released to automate their reconstruction process *de novo*. In order to enable gene deletion simulations and integration of gene expression data, these networks must include gene-protein-reaction (GPR) rules, which describe with a Boolean logic relationships between the gene products (e.g., enzyme isoforms or subunits) associated with the catalysis of a given reaction. Nevertheless, the reconstruction of GPRs still remains a largely manual and time consuming process. Aiming at fully automating the reconstruction process of GPRs for any organism, we propose the open-source python-based framework GPRuler. By mining text and data from 9 different biological databases, GPRuler can reconstruct GPRs starting either from just the name of the target organism or from an existing metabolic model. The performance of the developed tool is evaluated at small-scale level for a manually curated metabolic model, and at genome-scale level for three metabolic models related to *Homo sapiens* and *Saccharomyces cerevisiae* organisms. By exploiting these models as benchmarks, the proposed tool shown its ability to reproduce the original GPR rules with a high level of accuracy. In all the tested scenarios, after a manual investigation of the mismatches between the rules proposed by GPRuler and the original ones, the proposed approach revealed to be in many cases more accurate than the original models. By complementing existing tools for metabolic network reconstruction with the possibility to reconstruct GPRs quickly and with a few resources, GPRuler paves the way to the study of context-specific metabolic networks, representing the active portion of the complete network in given conditions, for organisms of industrial or biomedical interest that have not been characterized metabolically yet.

## Author summary

Over years, several methodologies have been proposed to integrate omics data into metabolic models in order to derive context-specific networks that represent the active portion of the network under specific conditions. In this way, biologically meaningful phenotypic predictions can be derived as a function of genes expression profiles encoding for subunits or isoforms of the involved enzymes. Regardless of the used approach to integrate omics data, the reliability of the formulated hypotheses strongly depends on the quality of gene-protein-reaction (GPR) rules included into the models, which describe how gene products concur to catalyze the associated reactions. To date, the reconstruction of GPR rules for their integration within metabolic networks still remains a largely manual and time consuming process. Therefore, we propose the open-source framework GPRuler to automate the reconstruction process of GPR rules for any living organism. Applying the developed tool to four case studies, we verified the ability of GPRuler to reproduce the original GPR rules with a very high level of accuracy. Moreover, in all the tested scenarios, the proposed approach revealed to be in many cases more accurate than the original models.

## Introduction

Current advances in genome sequencing technologies enable a fast and cheap overview into the genetic composition of virtually any organism. Nevertheless, determining the global metabolic profile of a cell or organism is fundamental to provide a comprehensive readout of its functional state, resulting from the interplay between genome, biochemistry and environment. In this context, genome-scale metabolic models (GEMs) offer a systemic overview for the investigation of cell metabolic potential, because of their key feature of embracing all available knowledge about the biochemical transformations taking place in a given cell or organism [1].

Over the years, extensive efforts have been made to automate the reconstruction process of these models, reaching high levels of quality and detail. However, less attention has been dedicated to clarify the associations among the set of genes involved in the catalysis of a given reaction.

According to the underlying catalytic mechanism, metabolic reactions can be classified as non-enzymatic and enzymatic. In the first scenario, metabolic reactions occur either spontaneously or catalyzed by small molecules, implying that no gene is necessary for their catalysis. In the second case, enzymatic reactions occur only when catalyzed by specific enzymes, which are protein macromolecules that are specifically responsible for reactions catalysis [2]. From the structural point of view, an enzyme may be classified as either monomeric or oligomeric entity. In its monomeric state, an enzyme consists of a single subunit, implying that a single gene is responsible of its final state. [3]. Conversely, in its oligomeric state, enzymes are protein complexes including multiple subunits that are all necessarily required to allow the corresponding reaction to be catalyzed. In general, enzymes differing in either their biological activity, regulatory properties, intracellular location, or spatio-temporal expression, may alternatively catalyze the same reaction and are known as isoforms [3].

Combining these two operators into expressions allow complex scenarios to be described, such as multiple oligomeric enzymes behaving as isoforms due to the sharing of a common part and to the presence of one or more subunits constituting distinctive features of the different isoforms.

**Fig 1:**
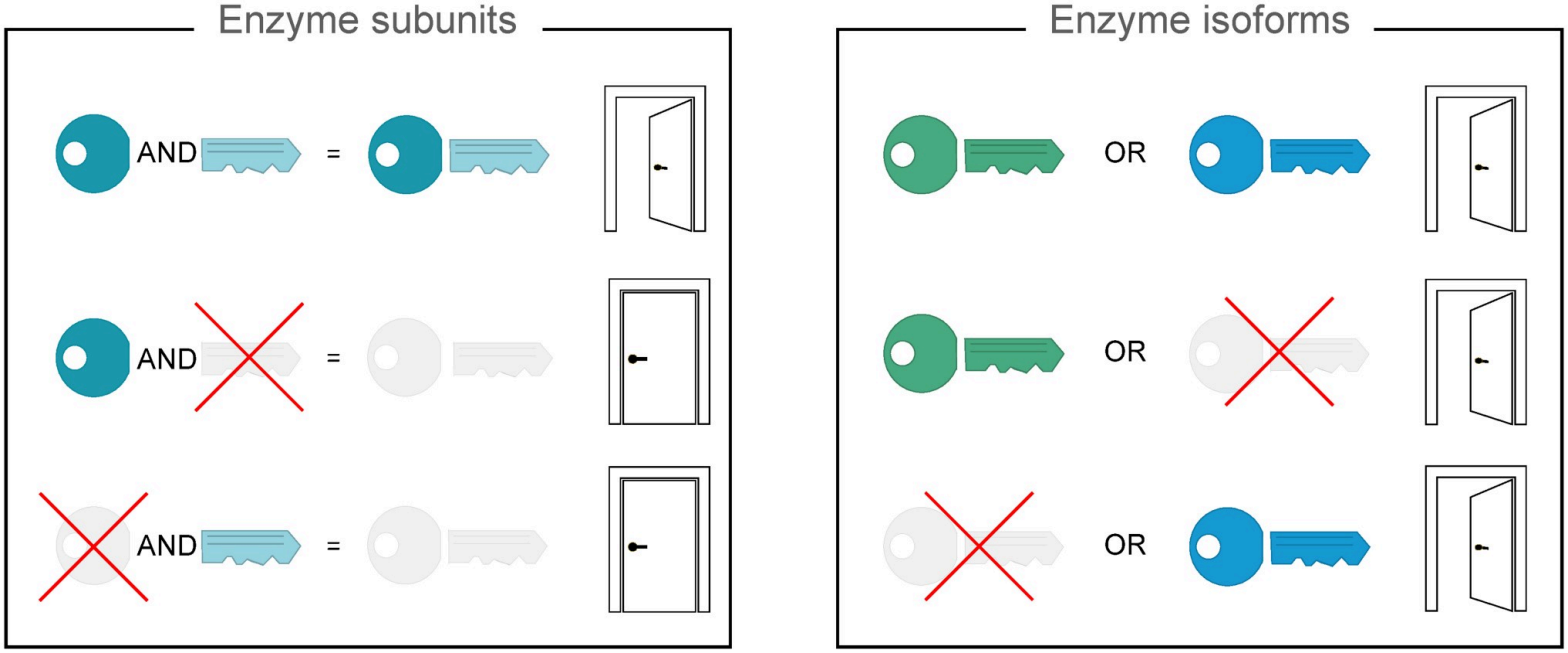
Logic of GPR rules. A metaphorical representation is exploited to explain the meaning of the AND and OR operators used in the reconstruction of GPR rules. In the box on the left named “Enzyme subunits”, the AND operator joins genes encoding for different subunits of the same enzyme. Metaphorically, the head and tail of the key represent the subunits of the same enzyme. Both parts of the key are required to open the door, and the lack of one of the two parts precludes the opening of the door. Biologically speaking, when both the subunits forming the enzyme are available, the enzyme can catalyse the reaction where it is involved. In the box on the right named “Enzyme isoforms”, the OR operator joins genes encoding for different isoforms of the same enzyme. In this case, two distinct keys representing the enzyme isoforms can alternatively open the door. Differently from the previous situation, just one of the two isoforms is sufficient to catalyse the reaction. Combining AND and OR operators, more complex scenario can be describe where both isoforms and subunits are involved.

In GEMs, the associations between genes, proteins and reactions and the description of how gene products concur to catalyze the associated reaction are usually encoded through logical expressions typically referred to as gene-protein-reaction (GPR) rules (Fig 1). GPR rules use the AND operator to join genes encoding for different subunits of the same enzyme, and the OR operator to join genes encoding for distinct protein isoforms of the same enzyme or subunit.

**Fig 2:**
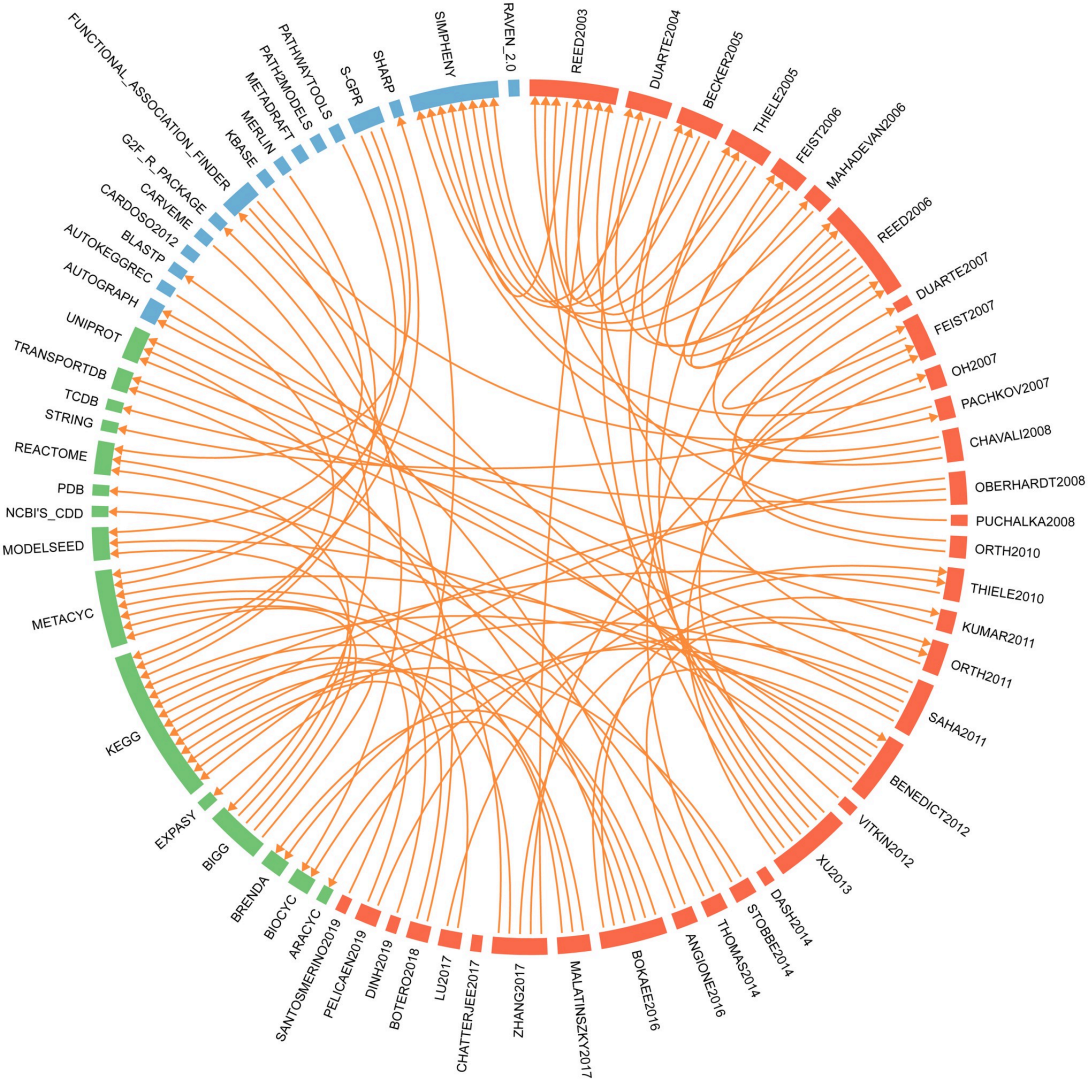
Literature analysis dealing with GPR reconstruction. In the circular plot, nodes correspond to the examined publications (red nodes), the adopted strategies (blue nodes) and data sources (green nodes). The nodes are ordered chronologically within the red category and alphabetically in the other two ones. Directed edges connect a given node to the to the exploited sources. If the source is not mentioned, the node remains isolated. Rectangles identify through their size the citation status of the corresponding node. References associated to each label are reported in the S1 File.

We performed a survey of literature on gene-protein-reaction rules and reconstructed a network of citing and cited papers to highlight the most adopted strategies and main data sources used, as reported in Fig 2. It can be observed that the sources exploited in literature are biological databases such as KEGG [4], UniProt [5], STRING [6] and MetaCyc [7], as well as genome annotations [8, 9], biochemical evidence presented in journal publications and reviews [10–12], and GPRs of closely related organisms [13]. The analysed publications also include work that relies exclusively on manual reconstruction of GPR rules, as e.g. [14], and work that limits to reconstruct one-to-one associations between genes and reactions rather than logical relationships among genes, proteins, and reactions [15].

The analysis of current formally defined pipelines for the automatic generation of GPR rules in metabolic reconstructions highlighted two different categories of tools. In the first one, we found tools able to reconstruct the association reaction-genes without determining the Boolean operators connecting them. Hence time-consuming manual contribution is required to obtain the final correct GPRs. Just to name one tool within this category, the *RAVEN* (Reconstruction, Analysis and Visualization of Metabolic Networks) *Toolbox 2.0* [16] is a MATLAB-based tool for constraint-based metabolic modelling [16] that assists de novo semi-automated draft model reconstructions for given target organisms starting from genome sequence, including the possibility to automatically reconstruct their GPR rules. The complete pipeline can be executed starting from the genome sequencing of a given organism.

In the second category, we found a number of tools dealing with the automated or semi-automated generation of both genes and Boolean operators of GPRs by generally using individual or a limited set of data sources. One of the most cited tool among them is *SimPheny* [17]. Simpheny is a closed source industrial software application developed by the Genomatica company, which allows for the creation, enrichment and analysis of genome-scale metabolic models. As detailed in [18], Simpheny makes use of BLASTP similarity search of genome sequences of the target organism against genes of several high-quality genome-scale metabolic models. In this way, however, only draft GPR rules are created and an intensive manual revision of Boolean operators is required using gene annotations and available biochemical and physiological information. Moreover, as shown in Fig 2, literature analysis revealed that the usage of Simpheny prevailed over a fairly limited time period.

An attempt to fully automatically reconstruct GPRs is also represented by the open source software Metabolic Models Reconstruction Using Genome-Scale Information (*merlin*) [19]. Its graphical interface guides the user along all the proposed features for genome-scale metabolic network reconstruction from genome annotation. The graphical interface allows users with no programming skills to use the tool. During our testing, the software succeeded in automatically creating GPR associations for the *Escherichia coli K-12* organism suggested in the tool documentation. Nevertheless, identification of the corresponding GPR associations relies exclusively on the KEGG BRITE database, from which information on the structure of the protein complex modules are retrieved. These rules are defined for sets of genes having similar roles, and conserved across several species using the KEGG ORTHOLOGY database. Although the valuable information available in KEGG database, its exclusive usage without including proteins structure and interactions coming from other already mentioned data sources could limit the outcomes.

Along similar lines, a number of other pipelines fall into this category, including Path2Models [20], ModelSEED [21], PathwayTools [22], kBase [23], CarveMe [24], MetaDraft [25], and foundational protocols as detailed in [26], which are based on genome annotation, experimental literature evidences or biological databases.

In view of the above, we propose a new methodology called GPRuler to efficiently automate the reconstruction process of GPR rules within metabolic networks of given organisms. In particular, we want to guarantee a white box among the currently available tools in order to introduce an open-source, clear and reproducible pipeline that can be applicable to any organism independently of its genome size, where the manual intervention is minimized in favour of an automatic information retrieval and managing. To achieve our aim, we rely on an extra available data source not yet exploited by state of the art, which is the Complex Portal [27] database, which contains information about protein-protein interactions and protein macromolecular complexes established by given genes.

## Materials and methods

### Tool implementation

GPRuler was implemented in Python programming language (version 3.7). The overall GPRuler pipeline can be executed starting from two alternative inputs. In the first case, an already available draft SBML model or a simple list of reactions lacking the corresponding GPR rules can be provided. In the second case, GPRuler takes as exclusive input the name of the organism of interest. In both cases, the inputs are firstly processed to obtain the list of metabolic genes associated with each metabolic reaction in the target organism/model. This intermediate output is then used as input for the core pipeline, which returns as ultimate output the GPR rule of each metabolic reaction.

The GPRuler tool tutorials are available in the qLS GitHub repository at https://github.com/qLSLab/GPRuler.

**Fig 3:**
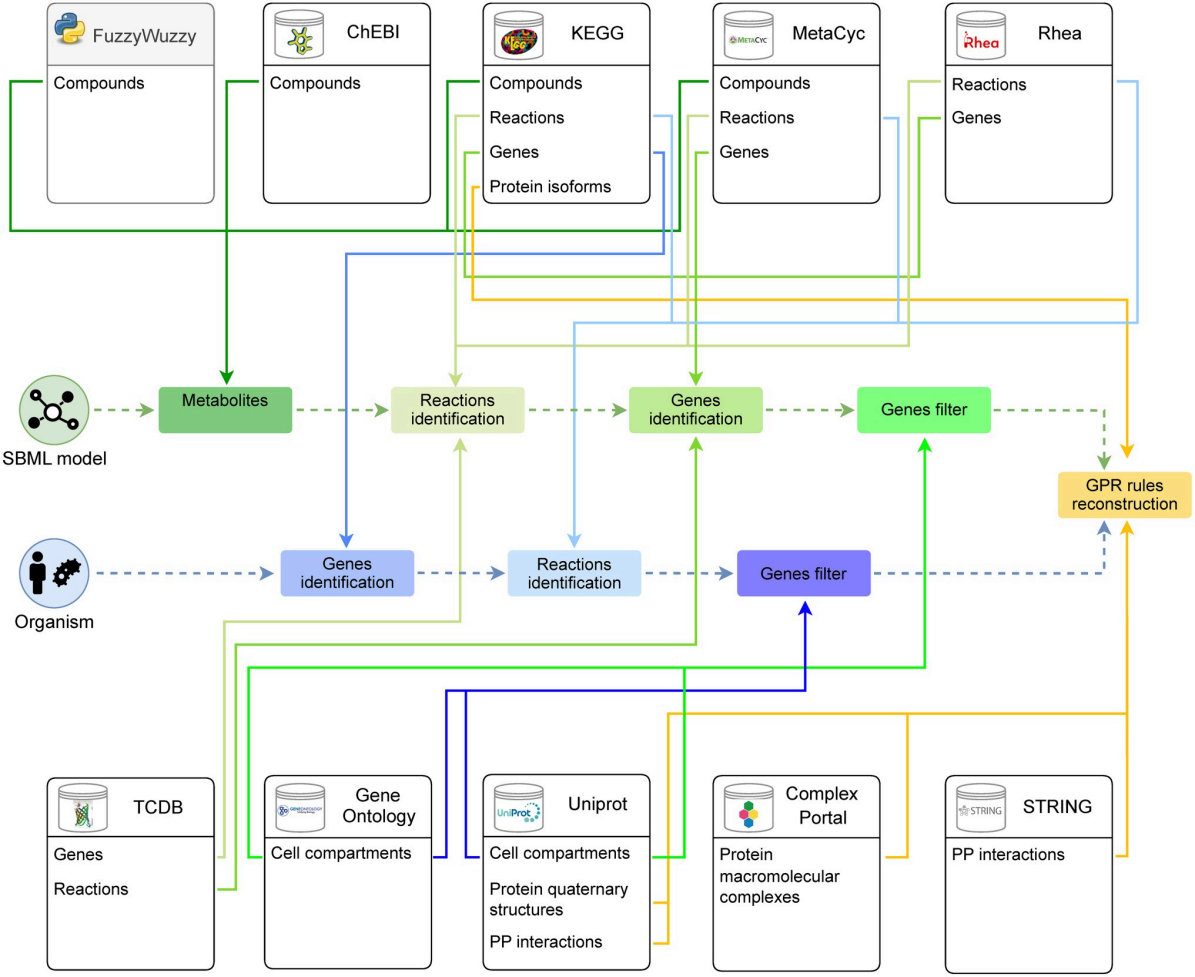
A detailed graphical representation of GPRuler tool. The central part of the figure illustrates the two alternative paths that can be followed to reconstruct the GPR rules according to the two possible inputs of GPRuler: the SBML model (green) and the organism name (blue). The green and the blue rectangles connected to each other by dashed arrows show the steps to follow in each path to achieve the core pipeline (orange rectangle), which returns as ultimate outcome the GPR rules. The ten boxes on the top and bottom of the figure represent the exploited data sources used by GPRuler, including both biological databases (white boxes) and the FuzzyWuzzy Python package (gray box), listing which information is retrieved from each of them. Each coloured arrow links each step of the pipeline to the used source and, in particular, to the type of data for which that particular source is queried.

The overall proposed pipeline and its sequential execution steps are schematized in the following and in Fig 3.

### From reactions list to annotated metabolic genes

GPRuler can handle the scenario where a metabolic model has already been reconstructed in terms of included biochemical reactions, but information on the associated genes and, of course, on GPR rules is not available yet.

In order to reconstruct this information, GPRuler queries five biological databases, namely MetaCyc, KEGG, Rhea [28], the Chemical Entities of Biological Interest (ChEBI) [29], the Transporter Classification Database (TCDB) [30]. Firstly, in order to speed up all the subsequent steps and, thus, the entire pipeline, we downloaded all data available in these databases regarding the target organism, in their most recent version in the following access dates: MetaCyc: December 16th 2020; KEGG: December 16th, 2020 for all the pipeline before data mining of gene relationships, February 8th, 2021 for data mining of gene relationships; Rhea: December 16th, 2020; ChEBI: December 16th, 2020; TCDB: December 19th, 2020.

To obtain the set of genes involved in the catalysis of a given input reaction, cross-links identifiers to the above mentioned databases must be retrieved. To this aim, GPRuler identifies *in primis* the metabolites involved in the reaction (Fig 3, Metabolites identification green box), by using the ChEBI database. ChEBI is an open source dictionary about any distinguishable molecular entity naturally or synthetically produced involved in living organisms processes. Additionally to all the provided information about name, identifier, synonyms and direct links to the entries in KEGG and MetaCyc databases, we exploited two other pieces of information obtained from ChEBI: the IUPAC International Chemical Identifier (InChI) and the ChEBI ontology. The InChi is an alphanumeric string providing a unique digital signature to any compound based on its structural representation. The ChEBI ontology is a tree-like description of structurally related compounds, which are included in increasingly more general categories.

Metabolites are also identified in our pipeline through the MetaCyc database, which is a curated database of experimentally derived metabolic pathways involved in both primary and secondary metabolism, and of their associated metabolites, reactions, and genes. The PythonCyc package provided a Python interface to Pathway Tools, which is bioinformatics software accessing to all the information available in MetaCyc database.

Finally, KEGG COMPOUND and KEGG GLYCAN databases are queried to retrieve data about all the compounds annotated in this database, including links to external database, such as ChEBI. KEGG is a biological resource consisting of multiple cross-linked databases that collect information relative to genes, enzymes, compounds, reactions and pathways.

Once the stored compounds data in ChEBI, MetaCyc and KEGG are extracted, the FuzzyWuzzy Python package is used for string matching between the target metabolite and a list of strings. Leveraging the Levenshtein distance to compute the similarity score between strings, the FuzzyWuzzy package extracts the best matches to the target string. After manual inspection of the string matching performances, we set an acceptance threshold for the metabolite name of 91, which was able to exclude clearly wrong matches.

Once the metabolites involved in each reaction of the input list have been assigned a proper identifier, GPRuler determines the reaction identifiers (Fig 3, Reactions identification green box), as annotated in KEGG REACTION, MetaCyc and Rhea databases. Rhea is an expert-curated biochemical reactions resource using the chemical dictionary from ChEBI to describe reaction players. Rhea is currently linked to several resources, including UniProtKB, KEGG and MetaCyc.

In view of the fact that the three considered reaction databases are not completely independent one from another, as first action before querying them, our pipeline joins them in what we refer to as macro database. This macro database includes the union of the biochemical reactions present in each of the examined databases. In case the same reaction is found in multiple resources (with the very same metabolites and stoichiometry), it is represented as a unique object encompassing all the available annotated information about the reaction. Each reaction entry will include the corresponding set of reactants and products, cross-links to external resources and the list of corresponding Enzyme Commission (EC) numbers and catalyzing genes, from each of the consulted databases.

The macro database is then queried to infer for each input reaction the corresponding identifier and hence the corresponding annotated list of genes (Fig 3, Genes identification green box). Target reactions can be splitted in two kinds: internal, i.e., taking place within a given compartment inside the model, and transport, i.e., moving metabolites across compartments. For internal reactions, to retrieve the identifier it is sufficient to search for a reaction in the macro database that shares the same reactants and products with the same stoichiometry.

To retrieve the identifier of transport reactions, it is necessary to query the TCDB database in addition to the macro database. The TCDB is a freely accessible database for transport proteins providing structural, functional, and evolutionary information about transporters from organisms of all types. This database relies on the Transporter Classification (TC) system, which is an approved classification system for membrane transport proteins analogous to EC system used for the enzymes, except that it also includes functional and phylogenetic information. Exploiting the mapping provided by TCDB between the TC systems and the substrates annotated to be transported, TC codes for the target transport reaction are identified and the corresponding list of genes is isolated.

Finally, we wanted to filter the identified list of genes associated with each reaction to retain only entries for which subcellular location is in accordance with the one reported in the input reaction list or SBML model (Fig 3, Genes filter green box). To this aim, annotations about gene products localizations are inferred from the Uniprot database and the Gene Ontology (GO) knowledgebase [31, 32]. GO provides an organism-independent description of genes and gene products in terms of biological processes involving them, associated molecular functions, and cellular components that refer to the cell location where a gene product is active. Each available annotation is associated with an evidence code indicating if the annotation was manually or computationally assigned to a particular term. In case of conflicting information, GPRuler gives priority to subcellular locations deriving from manual annotation over those deriving from automatic predictions, following the principle that the human (user) curated knowledge is more accurate than automatic one.

### From organism name to the annotated metabolic genes and reactions

When the input is the name of a target organism, GPRuler seeks to retrieve all annotated metabolic genes of this organism, by accessing the KEGG database resource.

The first step of GPRuler is the determination of the KEGG identification code corresponding to the target organism’s name typed by the user. The user may be asked to disambiguate among a list of possible candidates codes.

The unambiguous identification of the target organism is then exploited to look for the corresponding list of genes involved in the catalysis of metabolic reactions (Fig 3, Genes identification blue box). In particular, the KEGG GENOME database is firstly queried to retrieve the complete reference gene sequences of the target organism. In order to isolate only metabolic actors, the KEGG BRITE functional hierarchy is used to filter out all the non-metabolic genes, whereas the KEGG REACTION database is queried to extract from the remaining ones the corresponding list of biochemical reactions involving them (Fig 3, Reactions identification blue box). Programmatical access to all data stored in KEGG database is performed thanks to the Bioservices Python package [33]. To be as inclusive as possible, we also queried our macro database. Each reaction previously identified was then associated with the union set of the genes retrieved from the macro database and those previously associated with that reaction. Finally, the identified list of genes retrieved for each reaction is filtered according to their annotation of subcellular location from Uniprot and Gene Ontology, as already described (Fig 3, Genes filter blue box).

### Retrieving protein quaternary structures and protein-protein interactions from Uniprot

To characterize the relationships among the metabolic genes isolated in the previous steps of the pipeline and reconstruct the GPR rules (Fig 3, GPR rules reconstruction orange box), GPRuler relies on the information included in the Uniprot database (Access date: February 8th, 2021) and in particular in the “Interaction” section. Uniprot is an open-source database that provides a collection of manually and computationally determined functional annotations about proteins. Among the available data, the “Interaction” section organises in multiple subsections information about the quaternary structure of proteins and the set of binary protein-protein interactions established with other proteins or protein complexes. In particular, interchangeable participants and/or different chains or subunits belonging to the same complex are referenced through their gene names within a textual description.

For each metabolic gene associated with a reaction, GPRuler access the “Interaction” section of the Uniprot database to perform text mining, aiming at extracting, relatively to the input metabolic gene product, the list of genes participating in the same protein complexes, together with genes coding for isoforms. To guide the process of data extraction, we first identified the textual expressions containing relevant information. In this regard, we used the nltk Python library to tokenize the textual description of the “Interaction” section of investigated proteins and to carry out a frequency analysis, leaving aside the stop words that appeared frequently without conveying any meaning about the entire text. A frequency analysis of the remaining words highlighted the key words that, with the highest frequency count, guided the extraction of the target information [34].

In particular, the analysis of a random set of genes belonging to two of the most annotated and known organisms, namely *Homo sapiens* and the yeast *Saccharomyces cerevisiae*, revealed the following four terms as top words: complex, component, interact, and by similarity. These key terms allow for identification of protein-coding genes that participate in the same protein complex of the queried protein, or that establish binary protein-protein interactions with it. This information about protein-protein interactions reported in Uniprot derives from the IntAct Molecular Interaction Database [35] and originates from literature automatic mining or from manual submission, as well as from information propagated from experimentally characterized proteins in closely related species.

To enrich the results of the search for the target organism in the “Interaction” section of Uniprot database, in addition to the analysis of monograms, we analyzed different length n-grams. This allowed us to identify more extended expressions, such as “interact with”, “part of a complex with”, “consist of”, “associate with” and “heteromerization with”.

In addition to the “Interaction” section, GPRuler considers for text mining the “Function” section of the Uniprot database due to its role in describing isoform-specific functions or functionalities associated with the investigated protein as precursor of protein complexes. Information retrieval follows the same initial text processing described ahead.

GPRuler performs programmatic access to data stored in the Uniprot database via the Bioservices package.

### Retrieving protein macromolecular complexes from Complex Portal

In addition to textual annotation of proteins, Uniprot database also offers cross-references to other biological databases storing data about protein-protein interactions.

Among them, Complex Portal is a manually curated database of macromolecular complexes relative to 21 key organisms representative of different taxonomic groups, including *Homo sapiens*, *Mus musculus*, *Saccharomyces cerevisiae*, *Escherichia coli*, *Arabidopsis thaliana*, *Caenorhabditis elegans*. All the included data derive from both physical and molecular interaction evidences resulting from literature search and manual inference from scientific background or homolog information in closely related species. In order to extract all the components of protein complexes, GPRuler firstly extracts from Uniprot the identifiers of protein complexes formed by each input metabolic gene product, and then accesses the Complex Portal data (Access date: February 8th, 2021) by means of Python requests and json libraries in order to extract all the components constituting the investigated complexes.

### Retrieving known and predicted protein-protein interactions from STRING

Another cross-referenced database to Uniprot about protein-protein interactions is STRING database. STRING is a database with a high coverage of both annotated proteins and organisms. It deals with physical and functional associations of specific target proteins resulting from integration of publicly available protein–protein interaction data sources, including inference of functional associations between genes from genomic-context predictions, genome-scale laboratory experiments, gene co-expressions, co-citation analysis of scientific texts, manually curated interactions from other biological databases, knowledge transfer between organisms. The available knowledge is further complemented with computational predictions.

The programmatic access to STRING database (Access date: February 8th, 2021) is made possible by the STRING application programming interface (API). Among the implemented functionalities, “retrieving the interaction network” and “performing functional enrichment” are used to extract other protein-coding genes constituting a complex with the queried protein. In particular, the “retrieving the interaction network” method allows one to retrieve the interaction network established by the target protein. Once this network is obtained, the “performing functional enrichment” method allows to perform functional enrichment analysis for each one of the selected proteins by mapping them onto several databases, including Gene Ontology, KEGG pathways, UniProt Keywords, PubMed publications, Pfam domains, InterPro domains and SMART domains. Thanks to this method it is possible to select, from the proteins included in the extracted interaction network, those belonging to the same protein complexes of the input queried protein.

### Retrieving protein isoforms from KEGG

Another type of relationship among genes involved in a given reaction that must be reconstructed is the possible existence of protein isoforms. In this regard, KEGG Orthology database (Access date: February 8th, 2021), which belongs to the collection of databases included in KEGG database, is a database of functional orthologs, which have been defined based on their molecular functions by exploiting experimentally characterized genes or proteins with similar sequences in other organisms. From the KEGG gene identifier under investigation, it is possible to trace back to the associated KEGG Orthology identifier that returns for the specific organism under investigation the set of genes having this identifier, which are grouped together because of molecular functions similarity. Similarly to Uniprot, Bioservices package is used to programmatically access KEGG ORTHOLOGY database and automatically extract all the necessary information. In case that genes identifiers are not expressed as KEGG identifiers, a converter from external databases to KEGG database is provided by exploiting specific functionalities of Bioservices.

### Joining all retrieved information to generate final GPR rules

In the last step of GPRuler, all the data retrieved in the previous four steps is used to list, for each queried gene, the genes coding for subunits of the same protein complex to which the target gene belongs to or for alternative protein isoforms. Once this information is available, GPRuler organizes it to generate the final GPR rule. In this regard, all the possible pairs of genes associated to a given reaction are linked with either an AND or OR operator according to the type of relationship between them that has been previously inferred by GPRuler. In particular, the AND operator is assigned if the two genes encode for different subunits of the responsible enzyme, whereas the OR operator is assigned if they encode for alternative protein isoforms of the associated enzyme. In the simplest scenarios all the involved genes encode for isoforms of the same enzyme or they all encode for distinct subunits of the same enzyme. In a more heterogeneous scenario, in which both enzyme subunits and isoforms are present, GPRuler firstly joins genes related by an AND operator and enclose them in separated parentheses and then all the information about genes encoding for protein isoforms is included.

## Results

The core ability of GPRuler is to determine the GPR rule associated with each reaction. It follows that determining the right relationships among the involved genes takes priority in validating the tool. To assess the degree of confidence of the reconstructed GPR rules and hence the accuracy of GPRuler, we exploited curated metabolic models as ground truth.

According to the type of relationships established among the genes involved in a given reaction, GPR rules can be categorized into five classes. In the simplest case, reactions are associated with empty rules (here labelled as “No gene” rules) when no gene is involved in reaction catalysis, or to single gene rules (here labelled as “One gene” rules) when a unique gene is responsible for reaction catalysis. In these cases, minimum effort from GPRuler is required because the final GPR rule will simply correspond to an empty string or to the unique responsible gene.

An active role of our approach is played over more complex situations when multiple genes are involved in reaction catalysis generating the here labelled as “Multi gene” rules, where the right relationships among the involved genes need to be determined. In particular, “Multi gene” GPR rules can be characterized exclusively by OR operators among genes (here labelled as “OR” rules), by AND operators (here labelled as “AND” rules), or by both AND and OR operators in the most intricate scenarios (here labelled as “Mixed” rules).

We executed GPRuler by using a workstation equipped with Intel Xeon 2.60GHz cpus.

### GPRuler performance evaluation via a comparison with ground truth GPR rules

Following the generation of the rules by GPRuler, we evaluated their accuracy by comparison with ground truth GPRs. We considered as ground truth the GPRs of four extensively curated models relative to well known organisms, namely HMRcore [36], Recon3D [37], Yeast 7 (version 7.6.0) [38] and Yeast 8 (version 8.3.4) [39]. To evaluate whether a rule reconstructed by GPRuler coincides with the corresponding ground truth rule, we compared their truth tables. The truth table consists of one column for each of the *N* Boolean input variables, for 2^*N*^ rows, one for each of their possible combination. The final column stores the evaluated value of the GPR for each row. We considered each pair of GPRs as identical only if their truth tables are identical. We labelled these identical cases as “Perfect match”. It is worth noting that comparison of rules exceeding 20 genes was performed manually because of the computational cost.

In the opposite case, when at least one row of the table differs (“negative match”), the discrepancy can be imputed either to differences in the set of genes involved in the two GPRs under comparison and/or to different Boolean operators connecting them. The first scenario can be excluded by means of the Jaccard index. The Jaccard index is defined as the cardinality of the intersection set divided by the cardinality of the union sets, and quantifies the gene coverage of each reconstructed GPR rule as compared to the ground truth. The Jaccard index ranges between 0, if any overlap is observed, and 1, when the two sets coincide. For reactions having Jaccard index of 1, we used normalized Hamming distance between the two truth matrices to quantify the mismatch extent of their GPRs in terms of the included operators. The normalized Hamming distance is the number of different positions in the two vectors over the length of the two vectors. It ranges between 0, if the two truth tables are opposite, and 1, if the two truth tables are identical.

**Fig 4:**
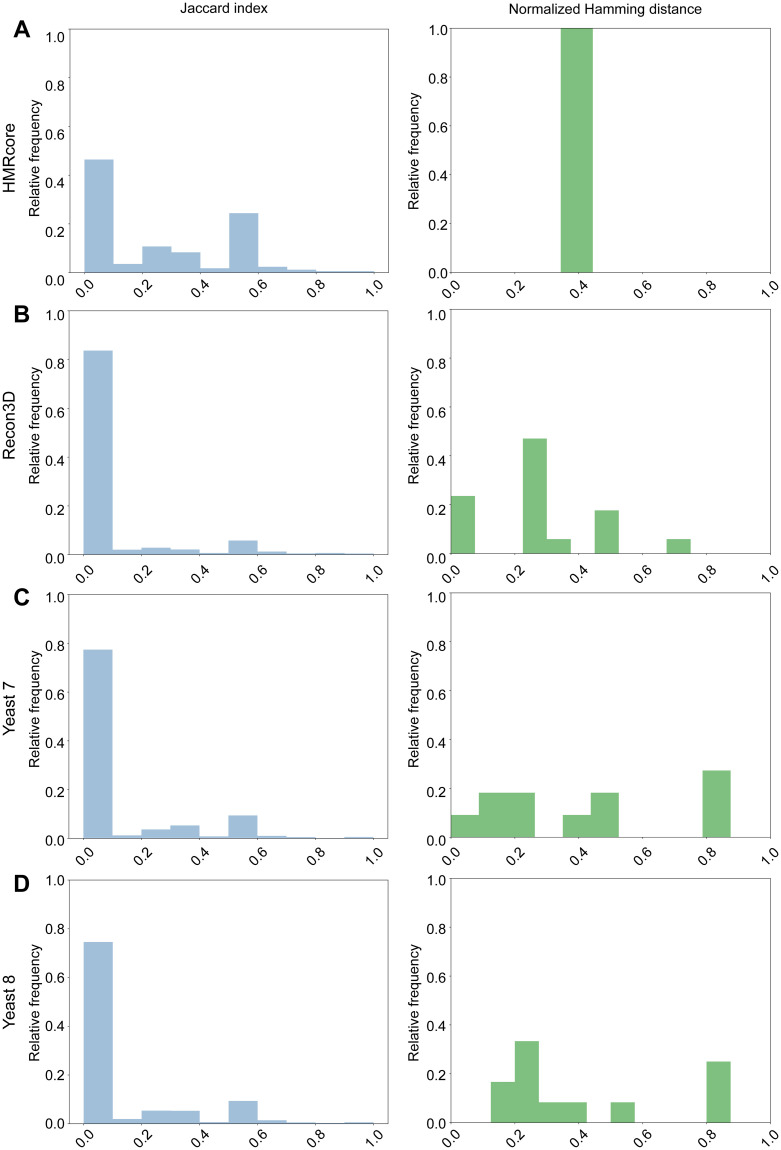
Evaluation of GPRuler performance in the obtained mismatches when compared with ground truth GPRs. The blue histograms on the left of each panel show the relative frequencies distribution of Jaccard indexes computed for the retrieved negative matches in all the four ground truth models. Specifically: HMRcore in Panel A; Recon3D in Panel B; Yeast 7 in Panel C; Yeast 8 in Panel D. We reported in the green histograms on the right of each panel the normalized Hamming distance between the two truth matrices of negative matches having Jaccard index of 1.

In Fig 4, we reported the Jaccard index computed for all the retrieved negative matches. As revealed by the distribution of their Jaccard index in all the four ground truth models, we obtained mostly low index values and very few cases with a Jaccard index of 1. This implies that the discrepancy in the two negative match GPRs is almost always due to differences in the set of involved genes. The complete list of Jaccard indexes and Hamming distances obtained for each tested model are included in the S2–S5 Files. Together with the reaction specific scores, we also computed a global Jaccard index to quantify the general extent at which expected genes are present through all reactions. The previously discussed prevalence of low values of Jaccard index in the negative matches is reflected in a low global Jaccard index for the four ground truth models. In detail, we obtained a Jaccard index of 0.36 in HMRcore, 0.55 in Recon3D, 0.63 in Yeast 7 and 0.65 in Yeast 8.

**Fig 5:**
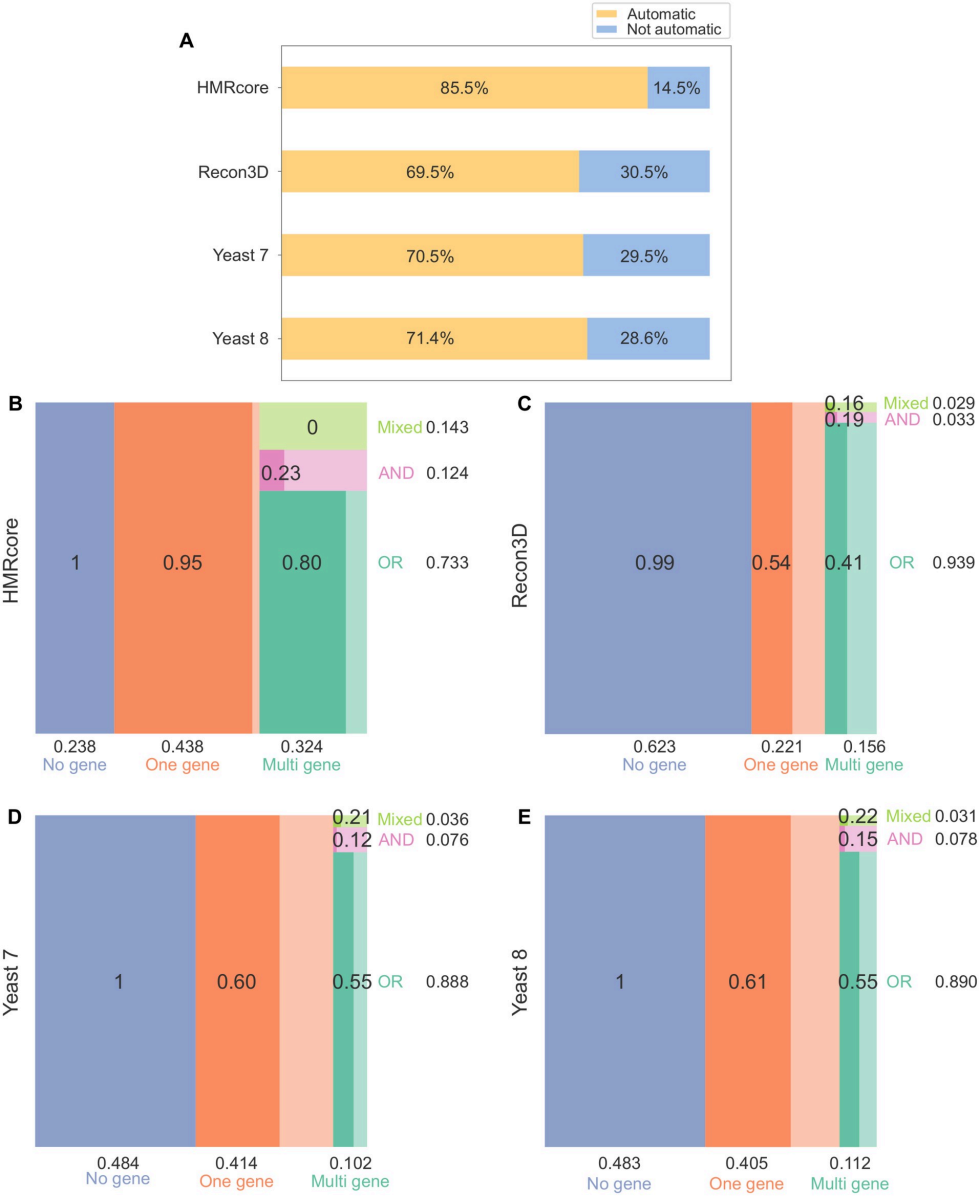
Assessment of GPRuler performance in reconstructing GPRs of ground truth models. Panel A shows a summary of GPRuler performance highlighting the percentage of the automatically reconstructed rules in each ground truth models (labelled as “Automatic” and coloured in dark yellow) against those that cannot be correctly reconstructed unless a subsequent curation by the user (labelled as“Not automatic” and coloured in light blue). The mosaic plots below show in B) HMRcore, C) Recon3D, D) Yeast 7 and E) Yeast 8 model the frequency of “Automatic” GPR rules as proportional to the size of internal rectangles. The rectangle portions having low transparency corresponds to the “Not automatic” rules. GPRs are classified according to the type of relationships established among genes involved in reaction catalysis as “No gene”, “One gene” and “Multi gene”. In the “Multi gene” class, the three subclasses “OR”, “AND” and “Mixed” are also represented. On the horizontal side of each mosaic plot, the proportion of “No gene”, “One gene” and “Multi gene” Automatic GPR rules in each model is reported. On the vertical side of each mosaic plot, the same information is reported for the three classes “OR”, “AND” and “Mixed” over the percentage of Automatic Multi gene rules.

For all negative matches, we manually checked the annotations of the involved gene products available in the Uniprot database and in organism specific databases to determine whether the differences observed in the GPRuler outputs are more or less in line with the underlying biology, as compared to the GPRs used as ground truth. In detail, we adopted information coming from GeneCards [40] and HGNC [41] databases for HMRcore and Recon3D models, and The Saccharomyces Genome Database (SGD) [42] for Yeast 7 and Yeast 8 models. In case all the genes and relative interactions in the ground truth GPR were consistent with biological knowledge and was not properly accounted by GPRuler, we labelled the GPR as “Not automatically reconstructed by GPRuler”. On the contrary, if some of the genes in the ground truth rule are wrongly associated to the reaction or improper Boolean operators are used between genes, whereas GPRuler rule is in line with the underlying biology, we labelled the GPR as “Corrected by GPRuler”. Finally, we considered as automatically reconstructed by GPRuler (“Automatic” labelled) both GPRs that were labeled as “Perfect match” and as “Corrected by GPRuler”. On the contrary, those rules that cannot be correctly reconstructed by GPRuler unless a subsequent curation by the user are referred to as “Not automatic”. The proportion of Automatic and Not automatic rules is shown in Fig 5A, respectively, as yellow and blue bars. In the following, we detailed the results obtained for the four ground truth models.

#### Performance evaluation of GPRuler on a manually curated core model

We firstly assessed the performance of GPRuler in reconstructing the GPR rules of HMRcore model, which is a core model of central carbon metabolism that we extracted from the genome-wide HMR metabolic model [43] and we subsequently introduced and curated in [36, 44–46]. We decided to use this model as ground truth to evaluate the performance of our approach because of the manually curated GPR rules associated to its reactions. Moreover, given the huge size of genome-scale models and the difficulty to control all the included GPRs, starting from a core model allowed a tighter control of the output.

The HMRcore model consists of 324 reactions that are mainly associated to single gene rules representing the 43.8%. The remaining rules are 23.8% classified as “No gene” and 32.4% as “Multi gene”.

The application of GPRuler on the reactions included in HMRcore and the subsequent comparison with those stored in the original model produced a perfect match for 158 of the 324 examined reactions, corresponding to a total of 48.8%. Considering the category of each rule, we correctly inferred 77.9% of “No gene” rules (60 out of a total of 77), 47.2% of the “One gene” rules (67 out of a total of 142), 15.4% of “AND” rules (2 out of a total of 13), 37.7% of the “OR” rules (27 out of a total of 86) (29 out of a total of 77), and none of the 15 “Mixed” rules.

Looking at the 166 wrongly obtained GPR rules (representing the 51.2% of total streactions of HMRcore reactions), 119 false negatives rules turned out to be replaceable with those generated through our methodology because in line with the information stored in the exploited databases. Following manual curation of the ground truth model, the percentage of correctly predicted GPR rules of HMRcore increased from 48.8% to 85.5 (as shown in Fig 5A), reaching the 100% of the GPRs that can be automatically constructed. In particular, we curated 17 rules afferent to the “No gene” category by increasing the corresponding coverage to 100% (77 out of a total of 77), 68 rules afferent to the “One gene” category by increasing the corresponding coverage to 95.1% (135 out of a total of 142), 1 rule afferent to the “AND” category by increasing the corresponding coverage to 23.1% (3 out of a total of 13), 33 rules afferent to the “OR” category by increasing the corresponding coverage to 80.5% (62 out of a total of 77), and none of the 15 “Mixed” rules. The remaining 14.5% of rules produced by GPRuler, depicted in Fig 5A as blue bar, corresponds to the cases labelled as “Not automatically reconstructed by GPRuler” and represents the obtained true mismatches. In Fig 5B, the frequency of Automatic rules is detailed for each class of GPRs.

The execution time of GPRruler on HMRcore was 2 hours and 24 minutes. The complete list of GPR rules generated by GPRuler for HMRcore model is available in the S2 File.

#### Performance evaluation of GPRuler on annotated genome-scale metabolic models

In light of the positive outcomes achieved with a small metabolic model of manually curated GPR rules, we extended the application of GPRuler at the genome-scale level for two of the most known and studied organisms, which are *Homo sapiens* and the yeast *Saccharomyces cerevisiae*. By considering the high level of knowledge of these organisms, we are confident in the accuracy and curation level of the GPR rules reconstructed for their metabolic reactions, whose generation derived from the integration of multiple biological databases, including SGD, BioCyc, Reactome, KEGG and UniProt.

The first considered genome-scale metabolic model is Recon3D, which is the most updated version of human metabolism. Recon3D model consists of 13543 reactions that are mainly associated to single gene rules representing the 43.3%. The remaining rules consist of 28.2% “No gene” rules and 28.5% “Multi gene” rules.

The application of GPRuler on the reactions included in this model and the subsequent comparison with those stored in the original model produced a perfect match for 4378 of the 13543 examined reactions, corresponding to a total of 32.3%. Considering the subcategory to which the obtained rules belong according to the included Boolean operators, we correctly inferred 60.7% of “No gene” rules (3565 out of a total of 5867), 13.3% of the “One gene” rules (507 out of a total of 3821), 1.6% of “AND” rules (4 out of a total of 254), 8.9% of the “OR” rules (299 out of a total of 3345), and 1.2% of the “Mixed” rules (3 out of a total of 256).

Looking at the remaining 9165 reactions (67.7%), 5039 rules turned out to be false mismatches and therefore replaceable in Recon3D model with those generated by GPRuler because in line with biological knowledge. After correcting the ground truth model, the percentage of correctly predicted GPR rules of Recon3D increased from 32.3% to 69.5% (as shown in Fig 5A), reaching the 100% coverage of the GPRs that can be automatically reconstructed. More in detail, we corrected 2301 rules afferent to the “No gene” category by increasing the corresponding coverage to 99.9% (5866 out of a total of 5867), 1574 rules afferent to the “One gene” category by increasing the corresponding coverage to 54.5% (2081 out of a total of 3821), 44 rules afferent to the “AND” category by increasing the corresponding coverage to 18.9% (48 out of a total of 254), 1081 rules afferent to the “OR” category by increasing the corresponding coverage to 41.2% (1380 out of a total of 3345), and 39 rules afferent to “Mixed” category by increasing the corresponding coverage to 16.4% (42 out of a total of 256). The remaining 30.5% of rules produced by GPRuler depicted in Fig 5A as blue bar, corresponds to the cases labelled as “Not automatically reconstructed by GPRuler” and represents the obtained true mismatches. In Fig 5C, the frequency of Automatic rules is detailed for each class of GPRs.

A consideration about the examined rules in Recon3D model concerns some recurrent imprecisions that should be revised in the original model independently of their comparison with rules generated from our approach. A first representative case regards the two reactions labelled in Recon3D model as “2OXOADOXm” and “AKGDm”. GPR rules of these reactions did not result in completely accordance with the corresponding rule reconstructed through GPRuler due to the inclusion of the PDHX gene. A manual investigation of Human Cyc database [47] highlighted the affiliation of this gene to another protein complex different from the one involved in the reactions catalysis, which is the pyruvate dehydrogenase. Similarly, “*GLXO1*” reactions includes all genes corresponding to a different enzyme compared to the involved one. Consequently, inclusion of these genes in the current rules should be reviewed. A second and consistent set of reactions was found to have contradictory GPR rules due to AND and OR operators simultaneously joining same genes. On behalf of this scenario, the reactions of Recon3D model labelled as “AHEXASE2ly”, “AHEXASEly”, “NDPK10”, “NDPK1” and “PFK” fall in this category. The most likely explanation of this behavior is the non proper automatic generation of GPR rules. Alongside with this hypothesis, a biological explanation of contradictory genes relationships might emerge in relation to alternative forms of the same enzyme. In these situations, the usage of parentheses within the logical expression becomes fundamental to discriminate automatic from biological annotations.

A last scenario emerged from the analysis of Recon3D model regards the reactions labelled as “GUACYC”, “CREATt4_2_r”, “GLYKm”, “GUACYC”, “HPCLxm”, “NOS1”, “NOS2”, “r0400”, “r0924”, “MTHFC”, “PSP_L”, “TPI”, “r0345”, “GLYK”, “FTHFL”, “MTHFD2”, “HMR_2041”, and “HMR_4790”. In this case, comparison of the original GPR rule with GPRuler indicated the need for a revision of the included gene identifiers. In all these reactions, genes that no longer exist due to dismissal of their identifiers are included, preventing the correct determination of the rule under consideration.

The execution time of GPRuler on Recon3D model was 231 hours and 33 minutes. The complete list of GPR rules generated by GPRuler for Recon3D model is available in the S3 File.

To further evaluate the performance of GPRuler, we considered as ground truth two other genome-scale models, namely Yeast 7 and Yeast 8, which are the two latest releases of *S. cerevisiae* genome-scale metabolism. The application of the pipeline to these two models returned comparable results.

Yeast 7 consists of 3493 reactions whose GPR rules resulted mostly categorized as “No gene” (34.1%) and “One gene” (48.7%). The remaining 17.2% consists of “Multi gene” rules. GPRuler applied to the reactions included in Yeast 7 proved to be able to correctly reconstruct 1600 GPR rules, representing the 45.8% of the rules in the model. Considering the category of each rule, we correctly inferred 69.6% of “No gene” rules (829 out of a total of 1191), 40.6% of the “One gene” rules (691 out of a total of 1701), 7.1% of “AND” rules (11 out of a total of 156), 17.1% of the “OR” rules (69 out of a total of 403), and none of the “Mixed” rules.

Thanks to a manual curation of the negative matches in the ground truth model, the global performance of GPRuler increased up to 70.5% (as shown in Fig 5A), totally covering the percentage of rule that can be automatically generated. The curation process of these 1893 rules revealed 861 of them as false mismatches and therefore better in line with the biological reality. More in detail, we corrected 362 rules afferent to “No gene” category by increasing the corresponding coverage to 100% (1191 out of a total of 1191), 328 rules afferent to “One gene” category by increasing the corresponding coverage to 59.9% (1019 out of a total of 1701), 8 rules afferent to “AND” category by increasing the corresponding coverage to 12.2% (19 out of a total of 156), 154 rules afferent to “OR” category by increasing the corresponding coverage to 55.3% (223 out of a total of 403), and 9 rules afferent to “Mixed” category by increasing the corresponding coverage to 21.4% (9 out of a total of 42). The remaining 29.5% of rules produced by GPRuler depicted in Fig 5A as blue bar, corresponds to the cases labelled as “Not automatically reconstructed by GPRuler” and represents the obtained true mismatches. In Fig 5D, the frequency of Automatic rules is detailed for each class of GPRs.

The execution time of GPRuler on Yeast 7 model was 14 hours and 2 minutes. The complete list of GPR rules generated by GPRuler for Yeast 7 model is available in the S4 File.

The updated model version of Yeast 7, namely Yeast 8, is a little bit larger than its predecessor since it consists of 3991 reactions. GPR rules classification showed that “No gene” and “One gene” rules cover, respectively, 34.5% and 47.4% of all the metabolic reactions included into the model, and “Mixed” rules cover the 18.1%. The execution of GPRuler perfectly reconstructed 1182 GPR rules, representing the 47.2% of the rules in the model. Considering the category of each rule, we correctly inferred 74.9% of “No gene” rules (1032 out of a total of 1377), 39.7% of the “One gene” rules (751 out of a total of 1892), 5.5% of “AND” rules (9 out of a total of 163), 17.5% of the “OR” rules (90 out of a total of 513), and none of the “Mixed” rules.

The manual curation of negative matches revealed, similarly to Yeast 7, the ability of GPRuler to correctly reconstruct the 71.4% of the model rules (as shown in Fig 5A), covering the 100% of rules that can automatically generated. Going into detail of the obtained GPRs, we correctly inferred, as false mismatches, 345 rules afferent to “No gene” category by increasing the corresponding coverage to 100% (1377 out of a total of 1377), 402 rules afferent to “One gene” category by increasing the corresponding coverage to 60.9% (1153 out of a total of 1892), 16 rules afferent to “AND” category by increasing the corresponding coverage to 15.3% (25 out of a total of 163), 194 rules afferent to “OR” category by increasing the corresponding coverage to 55.4% (284 out of a total of 513), and 10 rules afferent to “Mixed” category by increasing the corresponding coverage to 21.7% (10 out of a total of 46). The remaining 28.6% of rules produced by GPRuler depicted in Fig 5A as blue bar, corresponds to the cases labelled as “Not automatically reconstructed by GPRuler” and represents the obtained true mismatches. In Fig 5E, the frequency of Automatic rules is detailed for each class of GPRs.

The execution time of GPRuler on Yeast 8 model was 13 hours and 30 minutes. The complete list of GPR rules generated by GPRuler for Yeast 8 model is available in S5 File.

### GPRuler accuracy evaluation via comparison with simulation outcomes

To additionally evaluate the accuracy of GPRuler, we compared results of flux balance analysis simulation with experimental data. Specifically, we exploited the curated large scale phenotype annotations available in the SGD database for all genes of *Saccharomyces cerevisiae*. SGD reports for each gene whether the corresponding knock-out mutant is viable. In the opposite case, the gene is essential for the cell.

To mimic these experiments, we performed standard single gene deletion analysis used in flux balance analysis studies [48], by means of COBRApy. For each *in silico* single gene deletion, the optimal biomass production rate is calculated and compared to the wild-type model value. A gene is considered as “viable”, in case of positive biomass flux value, or “not viable”, in case of null biomass flux value.

We performed such analysis for the Yeast 8 genes when the model includes either the original GPRs or those reconstructed by GPRuler. We evaluated the accuracy of the two models, by comparing their viability predictions with information in SGD.

**Fig 6:**
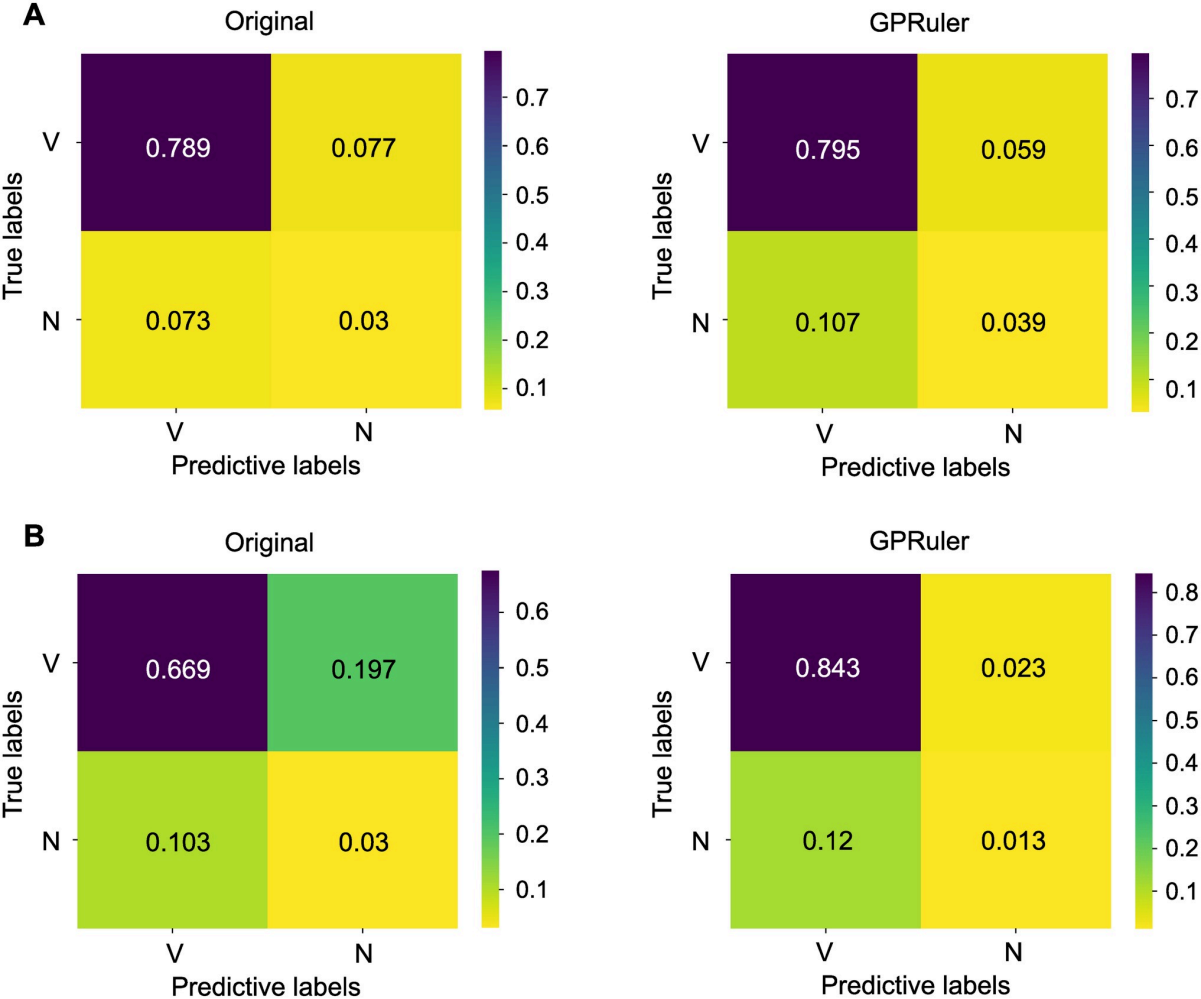
Evaluation of GPRuler performance by a comparison with *in silico* deletions of Yeast 8 genes. Panel A shows on the left the confusion matrix resulting from the simulation of all the genes of the original model (labelled as “Original”), and on the right the confusion matrix created from the Yeast 8 GPRs reconstructed by GPRuler (labelled as “GPRuler”). Panel B shows the confusion matrix in the “Original” (on the left) and “GPRuler” (on the right) model when only genes involved in Yeast 8 reactions classified as “Corrected by GPRuler” are considered. The two labels V and N correspond, respectively, to the “Viable” and “Not viable” phenotype. Each cell of the confusion matrix is coloured according to the relative frequency of the corresponding case, which is shown in the middle of the cell, following the color scale reported on the right of each plot.

The results of the analysis are visualized in Fig 6 in the form of a confusion matrix. As shown in Panel A of Fig 6, the Yeast 8 model reconstructed by GPRuler performs similarly to the original ground truth rules. The accuracy is indeed of 0.834 and 0.849, respectively. Furthermore, if we just consider genes involved in Yeast 8 reactions classified as “Corrected by GPRuler”, whose confusion matrices are shown in Panel B of Fig 6, the accuracy is far better in the Yeast 8 model reconstructed by GPRuler than in the original one. The accuracy is indeed of 0.856 and 0.699, respectively. This outcome confirms the ability of GPRuler to curate the original GPR rules.

## Discussion

Regardless of the starting input used from GPRuler to reconstruct GPR rules, the accuracy of the generated output mainly depends on the level of annotation of the investigated organism within public biological databases.

Two main classes of errors emerged from the analysis of our results. The first class regarded the Uniprot database and the textual annotation of gene products it provides. This database groups any biological knowledge about a given gene product spanning from the general function to the annotated catalytic activity, to cross-references pointing to data collected from external databases. Moreover, a lot of the available information is hidden behind the annotated GO terms, which provide a textual description about biological processes and molecular functions associated to the gene product. All these data need to be explored to infer the correct biological function and biochemical reactions that gene products catalyse in order to solve the association between genes and reactions and how genes interact among them. However, analyses on text structures and on how information about protein function, structure and interactions is stored in the database revealed a lack of uniformity in the way data are stored and presented. This issue sometimes led to problems of failed mining of gene products annotations with the consequent lacking of the correct information within the generated rules.

Most of GPRuler failures arose from the impossibility to correctly codify upstream of the pipeline the associations between reactions and genes. Due to insufficient availability of data within the here exploited biological sources, GPRuler did not allow for an automated retrieval of these data, preventing from reconstructing the correct rule. In this context, Uniprot and Gene Ontology and the large quantity of information they contain could help to infer these relationships and generate the correct rules. Nevertheless, due to the lack of structured data in these databases, more work is required to process and understand the provided information. For this reason, sophisticated text mining algorithms will be evaluated for a more efficient data retrieval and interpretation. In addition, the success of GPRuler execution is also invalidated from the fact that much of the information we are looking for to reconstruct the right relationships are stored in a variety of organism-specific databases that we do not include in GPRuler given out intent of developing an organism-independent pipeline. Consequently, only biological databases that are generic for all domains of life have been considered.

In the light of the above and as a consequence of the missing detailed description of the pipeline followed in the here tested ground truth models to generate the included GPR rules, there is reason to believe that most of the included data mainly come from manual curation.

The pipeline has been conceived to be completely automatic, minimizing the manual contribution. When GPRuler starts from an existing metabolic model, how information is stored within models becomes crucial for the correct success of the tool. This specifically refers to the presence within the tested models of metabolites not always automatically traceable to the corresponding object into the considered biological databases, because of a missing standard in the used nomenclature that prevents from finding any link to existing compounds database. This issue pushed us to draw up a dedicated strategy in order to automatically identify metabolites involved in a given list of reactions. Nevertheless, due to the reasons explained above, this automatic procedure required in few specific cases a feedback by the user to review metabolites for which a shortlist of candidate identifiers is returned. In other situations, a manual curation of some particular instances has been instead necessary. An example is represented by the metabolite *ACP1* that corresponding to the Acyl-carrier protein should be included as *ACP* because it does not exist in the indicated form. Furthermore, we noticed that, within the tested models, reactions involving the NADPH cofactor are linked in some specific cases to reactions objects that within biological databases involve the adrenal ferredoxin metabolite. A careful review of biological literature revealed that the activity of the adrenal ferredoxin depends on NADPH [49]. Therefore, we manually added ferredoxin to the list of identifiers associated to NADPH. Similarly, the electron transferring-flavoproteins, which are FAD-containing proteins [50], have been linked to the FAD and FADH_2_ cofactors. Finally, the last two cases of revised metabolites regarded lack of the oxidation state of a given metabolite that may result in an ambiguity of its identification, such as for the iron element that, usually existing as +2 or +3 cations, is used in Yeast 7 model to just indicate the ferrous ion form, and the usage in Yeast 7 and Yeast 8 models of the generic compound diglyceride for referring, as reported in KEGG COMPOUND database, to the specific chemical entity 1,2-Diacyl-sn-glycerol.

We remark that the Jupyter notebooks available in the GitHub of GPRuler make the pipeline fully reproducible. The user is guided through the few steps that require a manual intervention by interactive elements.

In addition to the scenarios mentioned above, cases of impossibility to identify metabolites by any method, either automatic or manual, occurred. Being the identification of metabolites the starting point of GPRuler, an insufficiently curated metabolic model becomes limiting for the correct detection of the related catalysing genes and their relationships, affecting in this way the performance of the tool. As discussed in S6 File, this phase of the pipeline will benefit in a future version of the tool from the integration of MetaNetX/MNXref [51], which provides cross-references between metabolites from major public biochemical databases and a selection of genome-scale metabolic networks. As reported in S6 File, the adoption of both MetaNetX/MNXref and our strategy can increase the coverage of identified metabolites, positively affecting the rest of the workflow.

When GPRuler starts from the organism name, we currently use the KEGG database to retrieve the list of reactions and the corresponding genes. The requirement for a genome annotation to be present in KEGG certainly limits the applicability of the method to only organisms included in the database itself. For this reason, a viable option in a future version of the tool will include an alternative approach based on the local or remote BLAST of the input protein sequences of the target organism rather than just exploiting their gene annotation, if available in KEGG. All the retrieved sequences from BLAST running will be processed in multiple databases, including KEGG, Reactome and MetaCyc, in order to get all the catalysed reactions. Furthermore, a parallel execution option will be evaluated in a future version of GPRuler to significantly reduce the computational time of the entire pipeline.

## Conclusion

In this work, we proposed an open-source tool called GPRuler in order to automate the reconstruction process of GPR rules for any living organism.

We applied the developed tool to four case studies, namely the HMRcore metabolic model and the genome-scale metabolic models Recon3D, Yeast 7 and Yeast 8, in order to assess the accuracy of GPRuler and the degree of confidence of the reconstructed GPRs.

By evaluating the resulting rules, as compared to their original counterparts, we verified the ability of GPRuler to reconstruct the GPR rules with a good level of accuracy. After manual inspection of the mismatches in the ground truth models, a large set of GPRs automatically reconstructed by GPRuler resulted closer to biological truth than original ones.

The developed pipeline, besides producing good outcomes in terms of performance and accuracy, allowed to point out some issues in the original GPR rules of ground truth models, especially in Recon3D, that need to be considered for future revisions of the models themselves. This makes GPRuler a viable companion to current tools within the same family.

With the advent of high-throughput technologies, an increasingly intensive generation of omics data occurred, paving the way towards the achievement of a system-level knowledge of cells. Although informative, relying only on these data is not enough to phenotypically characterize cells. In order to improve phenotypic predictions, it is appropriate to consider that a complete functional cell readout is not limited to gene expression analysis of cell. On the contrary, a characterization of its metabolic profiling, which represents the closest level of investigation to cell phenotype is required considering that a complex and non linear intracellular regulatory system occurs between gene expression and metabolic level.

Over years, several strategies have been proposed in order to integrate gene expression data into GEMs [46, 52–54] to derive context-specific networks representing the active portion of the complete network in given conditions. In this way, more biologically meaningful metabolic insights in distinct experimental conditions can be derived as a function of genes expression profiles encoding for subunits or isoforms of specific enzymes. Regardless of the approach developed to integrate omics data within GEMs, the success of these tools and the reliability of the formulated hypotheses strictly depends on the quality of the GPR rules included into the models. Hence, the importance of curating the GPR rules. Their good quality can also guarantee the successful outcome of multiple phenotype prediction experiments, including simulations of gene knock-out or gene expression variation, which allow to analyse the effect of these perturbations on the global system functioning.

Given the strong interest of life sciences research towards omics data integration, novel implements that improve new knowledge discovery by boosting the performance of already existing computational tools involved at the forefront in this research line currently represent a priority in the scientific community. GPRuler may complement all those metabolic reconstruction and data integration computational tools that would benefit, to minimize manual intervention, of a reliable implement able to reconstruct GPR rules fully automatically for any organism of interest. Although, as highlighted in the Results and Discussion sections, some aspects need to be improved, today GPRuler represents a significant step beyond the state of art in the GPRs reconstruction field, paving the way towards future versions of the pipeline.

## Supporting information

S1 File List of references associated to each label in Fig 2. (PDF)

S2 File GPR rules generated by GPRuler for HMRcore model. Each row shows in the column *Rxn* the reaction for which the GPR rule has been generated, in the column *rule_original* the corresponding original rule in the model, in the column *rule_GPRuler* the rule reconstructed by GPRuler and in the column *Evaluation* the the label assigned after comparison between the rule in the *rule_original* and in the *rule_GPRuler* column (*Perfect match*, *Corrected by GPRuler*, *Not automatically reconstructed by GPRuler*).
(CSV)

S3 File GPR rules generated by GPRuler for Recon3D model. Each row shows in the column *Rxn* the reaction for which the GPR rule has been generated, in the column *rule_original* the corresponding original rule in the model, in the column *rule_GPRuler* the rule reconstructed by GPRuler and in the column *Evaluation* the the label assigned after comparison between the rule in the *rule_original* and in the *rule_GPRuler* column (*Perfect match*, *Corrected by GPRuler*, *Not automatically reconstructed by GPRuler*).
(CSV)

S4 File GPR rules generated by GPRuler for Yeast 7 model. Each row shows in the column *Rxn* the reaction for which the GPR rule has been generated, in the column *rule_original* the corresponding original rule in the model, in the column *rule_GPRuler* the rule reconstructed by GPRuler and in the column *Evaluation* the the label assigned after comparison between the rule in the *rule_original* and in the *rule_GPRuler* column (*Perfect match*, *Corrected by GPRuler*, *Not automatically reconstructed by GPRuler*).
(CSV)

S5 File GPR rules generated by GPRuler for Yeast 8 model. Each row shows in the column *Rxn* the reaction for which the GPR rule has been generated, in the column *rule_original* the corresponding original rule in the model, in the column *rule_GPRuler* the rule reconstructed by GPRuler and in the column *Evaluation* the the label assigned after comparison between the rule in the *rule_original* and in the *rule_GPRuler* column (*Perfect match*, *Corrected by GPRuler*, *Not automatically reconstructed by GPRuler*).
(CSV)

S6 File Comparison between MetaNetX.org’s MNXref namespace and our methodology in identifying metabolite identifiers. (MD)
