# Supplementary material for: GPRuler: Metabolic gene-protein-reaction rules automatic reconstruction
Source: PLoS Comput Biol. 2021 Nov 8;17(11):e1009550. doi: 10.1371/journal.pcbi.1009550 (PMC8601613; doi:10.1371/journal.pcbi.1009550)
Supplement: S1 File — (PDF) [file pcbi.1009550.s001.pdf]

Table 1

This table reports the references associated to the labels present in figure 1.

Label	Reference	DOI
PUCHALKA2008	**Genome-scale reconstruction and analysis of the Pseudomonas putida KT2440 metabolic network facilitates applications in biotechnology.** PLoS computational biology, 4(10), 2008	10.1371/journal.pcbi.1000210
OH2007	**Genome-scale reconstruction of metabolic network in Bacillus subtilis based on highthroughput phenotyping and gene essentiality data.** Journal of Biological Chemistry, 282(39):28791–28799, 2007	10.1074/jbc.M703759200
STOBBE2014	Stobbe,M.D. et al. **Knowledge representation in metabolic pathway databases.** Briefings in bioinformatics, 15(3):455–470, 2014	10.1093/bib/bbs060
DINH2019	Dinh,H.V. et al. **A comprehensive genome-scale model for Rhodosporidium toruloides IFO0880 accounting for functional genomics and phenotypic data.** Metabolic engineering communications, 9:e00101, 2019. Elsevier.	10.1016/j.mec.2019.e00101
CHAVALI2008	Chavali,A.K., et al. **Systems analysis of metabolism in the pathogenic trypanosomatid Leishmania major.** Molecular systems biology, 4(1), 2008.	10.1038/msb.2008.15
VITKIN2012	Vitkin, E. and Shlomi, T. **MIRAGE: a functional genomics-based approach for metabolic network model reconstruction and its application to cyanobacteria networks.** Genome biology, 13(11):R111, 2012.	10.1186/gb-2012-13-11-r111
XU2013	Xu,Z., Sun,X. et al. **Construction and analysis of the model of energy metabolism in E. coli.** PloS one, 8(1), 2013. Public Library of Science.	10.1371/journal.pone.0055137
PACHKOV2007	Pachkov,M., et al. **Use of pathway analysis and genome context methods for functional genomics of Mycoplasma pneumoniae nucleotide metabolism.** Gene, 396(2):215–225, 2007.	10.1016/j.gene.2007.02.033
THOMAS2014	Thomas,A., et al. **Network reconstruction of platelet metabolism identifies metabolic signature for aspirin resistance.** Scientific reports, 4:3925, 2014.	10.1038/srep03925
MAHADEVAN2006	Mahadevan,R., et al. **Characterization of metabolism in the Fe(III)-reducing organism Geobacter sulfurreducens by constraint-based modeling.** Applied and Environmental Microbiology, 72(2):1558–68, 2006.	10.1128/AEM.72.2.1558-1568.2006
BECKER2005	Becker,S.A. and Palsson,B.Ø. **Genome-scale reconstruction of the metabolic network in Staphylococcus aureus N315: an initial draft to the two-dimensional annotation.** BMC microbiology, 5(1):8, 2005.	10.1186/1471-2180-5-8
ORTH2010	Orth,J.D. et al. **Reconstruction and use of microbial metabolic networks: the core Escherichia coli metabolic model as an educational guide.** EcoSal plus, 2005.	10.1128/ecosalplus.10.2.1

Label	Reference	DOI
THIELE2005	Thiele,I. et al. **Expanded metabolic reconstruction of Helicobacter pylori (iIT341 GSM/GPR): an in silico genome-scale characterization of single-and double-deletion mutants.** Journal of bacteriology, 187(16):5818–5830, 2005.	10.1128/JB.187.16.5818-5830.2005
PELICAEN2019	Pelicaen,R. et al. **Genome-scale metabolic reconstruction of Acetobacter pasteurianus 386B, a candidate functional starter culture for cocoa bean fermentation.** Frontiers in Microbiology, 10, 2019.	10.3389/fmicb.2019.02801
S-GPR	de Mas,I.M. et al. **Stoichiometric gene-to-reaction associations enhance model-driven analysis performance: Metabolic response to chronic exposure to Aldrin in prostate cancer.** BMC Genomics, 20(1):1–12, 2019.	10.1186/s12864-019-5979-4
CARDOSO2012	Cardoso,J. et al. **An Algorithm to Assemble Gene-Protein-Reaction Associations for Genome-Scale Metabolic Model Reconstruction.** IAPR International Conference on Pattern Recognition in Bioinformatics, 118–128, 2012.	10.1007/978-3-642-34123-6_11
G2F-R	Osorio,D. et al. **Find and Fill Gaps in Metabolic Networks.** R package	https://CRAN.Rproject.org/package=g2f
SHARP	Krishnakumar,S. et al. **SHARP: genome-scale identification of gene–protein–reaction associations in cyanobacteria.** Photosynthesis research, 118(1-2):181–190, 2013.	10.1007/s11120-013-9910-6
SIMPHENY	Price,N.D. et al. **Genome-scale microbial in silico models: the constraints-based approach.** Trends in biotechnology, 21(4):162–169, 2003.	10.1016/S0167-7799(03)00030-1
FAF	Green,M.L. and Karp,P.D. **Using genome-context data to identify specific types of functional associations in pathway/genome databases.** Bioinformatics, 23(13):i205–i211, 2007	10.1093/bioinformatics/btm213
BLASTP	Altschul,S.F. et al. **Gapped BLAST and PSI-BLAST: a new generation of protein database search programs.** Nucleic acids research, 25(17):3389–3402, 1997.	10.1093/nar/25.17.3389
AUTOGRAPH	Notebaart,R.A. et al. **Accelerating the reconstruction of genome-scale metabolic networks.** BMC bioinformatics, 7(1):296, 2006.	10.1186/1471-2105-7-296
AUTOKEGGREC	Karlsen,E. et al. **Automated generation of genome-scale metabolic draft reconstructions based on KEGG.** BMC bioinformatics, 19(1):467, 2018.	10.1186/s12859-018-2472-z
BIOCYC	Karp,P.D. et al. **The BioCyc collection of microbial genomes and metabolic pathways.** Briefings in bioinformatics, 20(4):1085–1093. 2019.	10.1093/bib/bbx085
TRANSPORTDB	Elbourne,L.D.H. et al. **TransportDB 2.0: a database for exploring membrane transporters in sequenced genomes from all domains of life.** Nucleic acids research, 45(D1):D320–D324. 2017.	10.1093/nar/gkw1068
BRENDA	Jeske,L. et al. **BRENDA in 2019: a European ELIXIR core data resource.** Nucleic acids research, 47(D1):D542–D549. 2019.	10.1093/nar/gky1048
REACTOME	Fabregat,A. et al. **The reactome pathway knowledgebase.** Nucleic acids research**, 46(D1):D649–D655. 2018.	10.1093/nar/gkv1351

Label	Reference	DOI
ARACYC	Mueller,L.A. et al. **AraCyc: a biochemical pathway database for Arabidopsis.** Plant physiolog, 2(2):453–460. 2003.	10.1104/pp.102.017236
UNIPROT	UniProt Consortium. **UniProt: a worldwide hub of protein knowledge.** Nucleic acids research, 47(D1):D506–D515. 2019.	10.1093/nar/gky1049
MODELSEED	Henry,C.S. et al. **High-throughput generation, optimization and analysis of genome-scale metabolic models.** Nature biotechnology, 28(9):977.	10.1038/nbt.1672
EXPASY	Gasteiger,E. et al. **ExPASy: the proteomics server for in-depth protein knowledge and analysis.** Nucleic acids research, 31(13):3784–3788. 2003.	10.1093/nar/gkg563
NCBI'S-CDD	Marchler-Bauer,A. et al. **CDD: NCBI's conserved domain database.** Nucleic acids research, 43(D1):D222–D226. 2015.	10.1093/nar/gku1221
TCDB	Saier Jr,M.H. et al. **The transporter classification database (TCDB): recent advances.** Nucleic acids research, 44(D1):D372–D379. 2016.	10.1093/nar/gkv1103
PDB	Berman,H.M. et al. **The Protein Data Bank.** Nucleic acids research, 28(1):235-242. 2000.	10.1093/nar/28.1.235
REED2003	Reed,J.L. et al. **An expanded genome-scale model of Escherichia coli K-12 (iJR904 GSM/GPR).** Genome biology, 4(9):R54, 2003.	10.1186/gb-2003-4-9-r54
REED2006	Reed,J.L. et al. **Towards multidimensional genome annotation.** Nature Reviews Genetics, 7(2):130, 2006.	10.1038/nrg1769
FEIST2007	Feist,A.M. et al. **A genome-scale metabolic reconstruction for Escherichia coli K-12 MG1655 that accounts for 1260 ORFs and thermodynamic information.** Molecular systems biology, 3(1), 2007.	10.1038/msb4100155
DUARTE2004	Duarte,N.C. et al. **Reconstruction and validation of Saccharomyces cerevisiae iND750, a fully compartmentalized genome-scale metabolic model.** Genome research, 14(7):1298–1309, 2004.	10.1101/gr.2250904
FEIST2006	Feist,A.M. et al. **Modeling methanogenesis with a genome-scale metabolic reconstruction of Methanosarcina barkeri.** Molecular systems biology, 2(1), 2006.	10.1038/msb4100046
ORTH2011	Orth,J.D. et al. **A comprehensive genome-scale reconstruction of Escherichia coli metabolism—2011.** Molecular systems biology, 7(1), 2011.	10.1038/msb.2011.65
ANGIONE2016	Angione,C. et al. **Multiplex methods provide effective integration of multi-omic data in genome-scale models.** BMC bioinformatics, 17(4):83, 2016.	10.1186/s12859-016-0912-1
BENEDICT2012	Benedict, M.N. et al. **Genome-scale metabolic reconstruction and hypothesis testing in the methanogenic archaeon Methanosarcina acetivorans C2A.** Journal of bacteriology, 194(4):855–865, 2012.	10.1128/JB.06040-11
SANTOSMERINO20[illegible]	Santos-Merino,M. et al. **New applications of synthetic biology tools for cyanobacterial metabolic engineering.** Frontiers in Bioengineering and Biotechnology, 7, 2019.	10.3389/fbioe.2019.00033

Label	Reference	DOI
DASH2014	Dash,S. et al. **Capturing the response of Clostridium acetobutylicumto chemical stressors using a regulated genome-scale metabolic model.** Biotechnology for biofuels, 7(1):144, 2014.	10.1186/s13068-014-0144-4
DUARTE2007	Duarte,N.C. et al. **Global reconstruction of the human metabolic network based on genomic and bibliomic data.** Proceedings of the National Academy of Sciences, 104(6):1777–1782, 2007.	10.1073/pnas.0610772104
MALATINSZKY2017	Malatinszky,D. et al. **A comprehensively curated genome-scale two-cell model for the heterocystous cyanobacterium Anabaena sp. PCC 7120**. Plant physiology, 173(1):509–523, 2017.	10.1104/pp.16.01487
OBERHARDT2008	Oberhardt,M.A. et al. **Genome-scale metabolic network analysis of the opportunistic pathogen Pseudomonas aeruginosa PAO1.** Journal of bacteriology, 190(8):2790–2803, 2008.	10.1128/JB.01583-07
ZHANG2017	Zhang,Y. et al. **A new genome-scale metabolic model of Corynebacterium glutamicum and its application.** Biotechnology for biofuels, 10(1):169, 2017.	10.1186/s13068-017-0856-3
BOTERO2018	Botero,K. et al. **A genome-scale metabolic model of potato late blight suggests a photosynthesis suppression mechanism.** BMC genomics, 19(8):863, 2018.	10.1186/s12864-018-5192-x
LU2017	Lu,H. et al. **Comprehensive reconstruction and in silico analysis of Aspergillus niger genome-scale metabolic network model that accounts for 1210 ORFs.** Biotechnology and bioengineering, 114(3):685–695, 2017.	10.1002/bit.26195
THIELE2010	Thiele,I. and Palsson,B.Ø. **A protocol for generating a high-quality genome-scale metabolic reconstruction.** Nature protocols, 5(1):93, 2010.	10.1038/nprot.2009.203
BOKAEE2016	Nazem-Bokaee,H. et al. **Assessing methanotrophy and carbon fixation for biofuel production by Methanosarcina acetivorans.** Microbial cell factories, 15(1):10, 2016.	10.1186/s12934-015-0404-4
SAHA2011	Saha,R. et al. **Zea mays iRS1563: a comprehensive genome-scale metabolic reconstruction of maize metabolism.** PloS one, 6(7), 2011.	10.1371/journal.pone.0021784
KUMAR2011	Kumar,V.S. et al. **Metabolic reconstruction of the archaeon methanogen Methanosarcina Acetivorans.** BMC systems biology, 5(1):28, 2011.	10.1186/1752-0509-5-28
CHATTERJEE2017	Chatterjee,A. et al. **Reconstruction of Oryza sativa indica genome scale metabolic model and its responses to varying rubisco activity, light intensity, and enzymatic cost conditions.** Frontiers in plant science, 8:2060, 2017.	10.3389/fpls.2017.02060
MERLIN	Dias,O. et al. **Reconstructing genome-scale metabolic models with merlin.** Nucleic acids research, 43(8):3899–3910, 2015.	10.1093/nar/gkv294
RAVEN2.0	Wang,H. et al. **RAVEN 2.0: A versatile toolbox for metabolic network reconstruction and a case study on Streptomyces coelicolor.** PLoS computational biology, 14(10):e1006541, 2018.	10.1371/journal.pcbi.1006541

Label	Reference	DOI
KEGG	Kanehisa,M. et al. **KEGG as a reference resource for gene and protein annotation.** Nucleic Acids Research, 44(D1):D457–62, 2016.	10.1093/nar/gkv1070
METACYC	Caspi,R. et al. **The MetaCyc database of metabolic pathways and enzymes-a 2019 update.** Nucleic Acids Research, 48(D1):D445–D453, 2020.	10.1093/nar/gkz862
STRING	Szklarczyk,D. et al. **STRING v11: protein–protein association networks with increased coverage, supporting functional discovery in genome-wide experimental datasets.** Nucleic acids research, 47(D1):D607–D613, 2019.	10.1093/nar/gky1131
PATH2MODELS	Büchel,F. et al. **Path2Models: large-scale generation of computational models from biochemical pathway maps.** BMC systems biology, 7(1):1-19, 2013.	10.1186/1752-0509-7-116
PATHWAYTOOLS	Caspi,R. et al. **The MetaCyc Database of metabolic pathways and enzymes and the BioCyc collection of Pathway/Genome Databases.** Nucleic acids research, 36(suppl_1):D623-D631, 2007.	10.1093/nar/gkm900
KBASE	Arkin,AP. et al. **KBase: the United States department of energy systems biology knowledgebase.** Nature biotechnology, 36(7):566-569, 2018.	10.1038/nbt.4163
CARVEME	Machado,D. et al. **Fast automated reconstruction of genome-scale metabolic models for microbial species and communities.** Nucleic acids research, 46(15):7542-7553, 2018.	10.1093/nar/gky537
BIGG	King,ZA. et al. **BiGG Models: A platform for integrating, standardizing and sharing genome-scale models.** Nucleic acids research, 44(D1):D515-D522, 2016.	10.1093/nar/gkv1049
METADRAFT	Hanemaaijer,M. et al. **Model-based quantification of metabolic interactions from dynamic microbial-community data.** PloS one, 12(3): e0173183, 2017.	10.1371/journal.pone.0173183
